# A randomised comparison of a four- and a five-point scale version of the Norwegian Function Assessment Scale

**DOI:** 10.1186/1477-7525-6-14

**Published:** 2008-02-15

**Authors:** Nina Østerås, Pål Gulbrandsen, Andrew Garratt, Jūratë Šaltytë Benth, Fredrik A Dahl, Bård Natvig, Søren Brage

**Affiliations:** 1 Section of Occupational Health and Social Insurance Medicine, Institute of General Practice and Community Health, Faculty of Medicine, University of Oslo, Norway; 2 Helse Øst Health Services Research Centre, Akershus University Hospital, Norway; 3 Faculty of Medicine, University of Oslo, Norway; 4 Institute of Health Management and Health Economics, University of Oslo, Norway

## Abstract

**Background:**

There is variation in the number of response alternatives used within health-related questionnaires. This study compared a four-and a five-point scale version of the Norwegian Function Assessment Scale (NFAS) by evaluating data quality, internal consistency and validity.

**Methods:**

All inhabitants in seven birth cohorts in the Ullensaker municipality of Norway were approached by means of a postal questionnaire. The NFAS was included as part of The Ullensaker Study 2004. The instrument comprises 39 items derived from the activities/participation component in the International Classification for Functioning, Disabilities and Health (ICF). The sample was computer-randomised to either the four-point or the five-point scale version.

**Results:**

Both versions of the NFAS had acceptable response rates and good data quality and internal consistency. The five-point scale version had better data quality in terms of missing data, end effects at the item and scale level, as well as higher levels of internal consistency. Construct validity was acceptable for both versions, demonstrated by correlations with instruments assessing similar aspects of health and comparisons with groups of individuals known to differ in their functioning according to existing evidence.

**Conclusion:**

Data quality, internal consistency and discriminative validity suggest that the five-point scale version should be used in future applications.

## Background

The measurement of functional ability is important in many contexts. While there often seems to be agreement as to the content of instruments for evaluation of function, there is relatively less consensus about the scaling of items. Item scaling vary in the number of response categories, the wording of category options and the use of all-point (where all categories are defined) or end-point (where only end-points are defined) scales [1,2]. The majority of health status and patient-reported outcome measures use all-point defined scales with between two and seven categories, the most popular being five-point scales including the agree/disagree Likert format. The generic Short Form 36-item (SF-36) Health Survey [3] uses five-point scales for seven of the eight health scales it includes. Other generic instruments such as the Nottingham Health Profile (NHP) [4] and EuroQol EQ-5D [5] use two- and three-point scales respectively. In the WHO Health and Work Performance Questionnaire, functional status is reported using different scales with between four and 11 points [6].

It has been argued that seven-point response scales are the maximum number that individuals are able to process [7] and some authors have advocated their use [8]. However, such scales are not widely used possibly because of the difficulty of finding suitable adjectives when seven all-point defined scales are used. Seven categories are also harder to fit across a page of A4 with a reasonably sized typeface. However, if the number of alternatives is less than the rater's ability to discriminate, the result may be a loss of information [2,9]. There is evidence that the reduction in reliability from ten to seven categories is quite small, but the use of five categories reduces the reliability by about 12 percent [2]. Hence it is argued that the minimum number of categories should be in the region of five to seven [2]. One review concluded that seven plus or minus two appears to be a reasonable range for the optimal number of response alternatives [9]. More recently, it was found that respondents preferences were highest for a ten-point scale followed by seven-point and nine-point scales [10]. The respondents rated scales with five, seven and ten response categories as relatively easy to use. Scales with two, three or four response categories were rated as relatively quick to use, but were unfavourable in terms of the extent to which they allowed the respondents to express their feelings adequately. If a scale does not allow respondents to express themselves, they may become frustrated or demotivated and the quality of their responses may decrease [10].

Previous research has shown that the greater the number of response options, the more reliable the scale is likely to be [11]. Simulations of categorization error have consistently shown that correlation between true values and scale scores increase with the number of response options [12]. Scales with relatively few response alternatives tend to generate scores with comparatively little variance, thereby limiting the magnitude of correlations with other scales [13,14]. The reduction in reliability is most severe for scales with four categories or less, but tends to level off once seven or more options are available. However, there is often a trade-off between scale reliability and ease of administration [11]. One study using the NHP indicated that the psychometric performance and patient acceptability was improved by using a five-point scale instead of the original shorter response format [15].

Following a recent systematic review, it was recommended that future research designs should allocate respondents to different versions of a questionnaire to compare approaches to item scaling [1]. Our study considered two different all-point defined scales using four and five response alternatives. The Norwegian Functional Assessment Scale (NFAS) was included in a large Norwegian population study on musculoskeletal pain, The Ullensaker Study 2004, to obtain self-reported levels of functional ability. Eligible persons were randomised to receive NFAS with the original four-point scale or a five-point scale.

The aim of this study was to compare the original four-point with the new five-point scale version by evaluating validity of the NFAS in a population. This will determine which version should be used in the future applications.

## Methods

### Study setting and sample

Ullensaker is a rural community which had 23,700 inhabitants in 2004. There are no major differences between the population of Ullensaker and the general population of Norway with respect to demographic characteristics [16]. In 2004, postal questionnaires, which included the NFAS along with questions relating to musculoskeletal pain, were sent to all 6108 inhabitants in Ullensaker municipality in the birth cohorts 1918–20, 1928–30, 1938–40, 1948–50, 1958–60, 1968–70 and 1978–80. Reminders were sent at eight weeks.

The sample was computer-randomised by an external company to either the four-point or the five-point scale version, herein referred to as the NFAS-4 and the NFAS-5. The Ullensaker Study questionnaire also included the Dartmouth COOP Functional Health Assessment Charts/WONCA(COOP/WONCA), General Health Questionnaire-20 (GHQ-20), Standardized Nordic Questionnaire, work ability, sickness absenteeism, and occupation.

The Regional Committee for Medical Research Ethics and The Norwegian Data Inspectorate approved the study.

### The Norwegian Function Assessment Scale (NFAS)

The Norwegian Function Assessment Scale (NFAS) is a self-report instrument developed by an expert group in social insurance in 2000 and is designed to assess the need for rehabilitation, adjustment of work demands among sick-listed persons as well as the rights to social security benefits [17]. The scale comprises 39 items derived directly from the activities/participation dimension in the International Classification of Functioning, Disability and Health (ICF) [18]. The items are relevant for assessing physical and mental functioning in working life, some relating to activities of daily living. The NFAS starts with the question "Have you had difficulty doing the following activities during the last week?" and respondents report 39 activities using a four-point scale: no difficulty, some difficulty, much difficulty, could not do it. The five all-point defined scale was developed to be more congruent with the qualifiers in the activities/participation dimension of ICF [19]: no difficulty, mild difficulty, moderate difficulty, much difficulty and could not do it.

Based on the results of principal component analysis from the previous study with sick-listed persons [17], the items form seven domains: Walking/standing (7 items), Holding/picking up things (8 items), Lifting/carrying (6 items), Sitting (3 items), Managing (7 items), Cooperation/communication (6 items), Senses (2 items). These domains have evidence for validity in sick listed persons [17]. The main application of the NFAS is likely to be social insurance. Hence it was decided to keep the domains from the earlier study with sick-listed persons [17]. It should, however, be anticipated that principal component analysis based on data from the general population in Ullensaker will yield somewhat different results. The first four and the last three domains are intuitively grouped into physical and mental domains respectively. Domain scores are calculated by adding the item scores and dividing by the number of items completed. NFAS total scores are calculated by adding all 39 item scores and dividing by the number of items completed. Low scores indicate good functional ability.

### COOP/WONCA

COOP/WONCA [20] is a generic health status measure, where functional status is self-reported with a time frame of the previous two weeks. It comprises six charts: Physical fitness, Feelings, Daily activities, Social activities, Overall health and Change in health. Each chart has five response alternatives with pictorial representations. The present study used an optional Pain chart in place of the Change in health chart.

### General Health Questionnaire (GHQ-20)

Psychological distress during the last two weeks was measured by the GHQ-20 [21], a widely used screening instrument for measuring non-psychotic psychiatric illness in a general population. Items are scored as the original GHQ score in a bi-modal fashion (0-0-1-1) [22].

Work ability was assessed by one question "To what degree is your ability to perform your ordinary work reduced today: hardly reduced at all, not much reduced, moderately reduced, much reduced and very much reduced" [23]. Respondents were asked to report whether they had experienced any pain or discomfort in ten different body regions during the previous week [24]. Sickness absenteeism was assessed by asking the respondents if they had been sick-listed during the previous year: no, less than 1 week, between 1–8 weeks, more than 8 weeks. Occupation was assessed with the categories: employed, housekeeping/full-time household work, unemployed, medical rehabilitation, disability pension, retired or student.

### Statistical analyses

#### Data quality

The two versions of the NFAS were compared for levels of missing data, and floor and ceiling effects, which were expressed as percentages.

#### Tests of scaling assumptions

Internal consistency was assessed by item-total correlation and Cronbach's alpha. Item-total correlation coefficients should meet 0.40 standard. Cronbach's alpha was considered acceptable for group comparisons when the coefficient exceeded 0.70 [25]. Item discriminant validity was assessed by analyzing correlations between the items and their domains (item-total) and between the items and the other domains (item-other) to see if the former was at least two standard errors higher than the latter, thereby indicating definite scaling success [26].

#### Construct validity

We hypothesised that scores from conceptually related domains of NFAS would correlate higher than scores of unrelated domains. We also hypothesised that NFAS scores would correlate higher with conceptually corresponding aspects of the COOP/WONCA, GHQ and Work Ability than with non-corresponding aspects. Correlation coefficients among measures of the same attribute should fall in the midrange of 0.40 – 0.80 [2].

It was hypothesised that those having a disability pension or rehabilitation benefit due to disease and those reporting being sick-listed previous year, would report lower functional ability. We also compared domain scores between those reporting musculoskeletal pain last week without mental distress (original GHQ score <4) and those with mental distress (original GHQ score ≥ 4) but no musculoskeletal pain. It was hypothesised that females, older persons and persons with shorter education would report lower functional ability than the males, younger persons and persons with longer education. Since data are categorical, non-parametric tests for independent samples were used to compare subgroups.

## Results

### Sample characteristics

Of the 6108 questionnaires posted, 3325 (54.4%) were returned. The response rate was lower for males (p < 0.001) and young or very old persons (p < 0.001) (Table 1). The response rates for the two versions were 54.0% for NFAS-4 and 54.8% for NFAS-5. 55 participants in birth cohort 1968–70 randomised to the NFAS-4 were erroneously mailed the NFAS-5 version. Hence, the subsamples differed significantly regarding age (p < 0.05), but not on any other background variables. Excluding the birth cohort 1968–1970 did not affect the results.

**Table 1:** Response rates by age and gender for the NFAS-4 and the NFAS-5 (N = 3325)

	**NFAS-4**		**NFAS-5**	
	**N (%)**	**Response rate %**	**N (%)**	**Response rate %**
**Females**	905 (55.9)	60.0	919 (53.9)	58.8
**Males**	715 (44.1)	48.0	786 (46.1)	50.8
**All**	1620	54.0	1705	54.8
				
**Age**:				
24–26	150 (9.3)	33.3	169 (9.9)	37.6
34–36	429 (26.5)	49.9	521 (30.6)	53.7
44–46	301 (18.6)	54.2	301 (17.7)	54.2
54–56	358 (22.1)	68.4	327 (19.2)	62.5
64–66	219 (13.5)	66.2	239 (14.0)	72.2
74–76	132 (8.1)	66.8	120 (7.0)	60.8
84–86	31 (1.9)	37.8	28 (1.6)	34.1

### Data quality

For respondents to the NFAS-4 and NFAS-5, there were no missing data for 78.5% and 82.4% respectively. All items had more missing data for the NFAS-4 than NFAS-5 (Table 2). The mean levels of missing data for individual items in the NFAS-4 and NFAS-5 were 3.3% and 2.6% respectively, which was statistically significant (p < 0.01). The same items within both versions had the highest percentage of missing values.

**Table 2:** Missing data, means and end effects for NFAS-4 and NFAS-5 items (N = 3325)

		**Missing %**		**Domain/item scores (mean)**		**Floor %**^a^		**Ceiling %**^a^	
		**NFAS-4**	**NFAS-5**	**NFAS-4**	**NFAS-5**	**NFAS-4**	**NFAS-5**	**NFAS-4**	**NFAS-5**
**Walking/standing**				**1.25**	**1.37**	**61.1**	**62.1**	**0.2**	**0.2**
Standing	1	3.0	2.6	1.19	1.29	84.9	83.2	0.3	0.2
Walking less than a kilometre on flat ground	2	4.6	3.5	1.19	1.30	87.5	84.3**	1.6	1.6
Walking than a kilometre on flat ground	3	3.8	2.8	1.32	1.44	80.6	79.1	4.3	3.2
Walking on different surfaces	4	3.6	3.3	1.24	1.35	81.0	80.1	0.8	0.7
Going up and down stairs	5	2.5	2.1	1.33	1.48	75.0	73.6	1.0	0.3*
Going shopping for your groceries	6	3.2	2.4	1.18	1.30	86.2	82.5**	0.6	1.0
Putting on your shoes and socks	7	1.9	1.8	1.21	1.36	81.6	78.1*	0.3	0.1
									
**Holding/picking up things**				**1.14**	**1.23**	**67.5**	**67.5**	**0.1**	**0.1**
Picking up a coin from a table with your fingers	8	2.5	1.9	1.10	1.17	91.6	89.5*	0.1	0.2
Holding and turning a steering wheel	9	5.3	4.9	1.06	1.13	96.3	93.3***	0.9	1.6
Driving a car	10	6.1	4.9	1.14	1.24	93.0	90.3**	3.2	4.1
Preparing food	11	2.5	2.0	1.10	1.16	92.3	89.9*	0.8	0.7
Writing	12	2.2	1.7	1.11	1.18	90.9	88.9	0.2	0.4
Performing everyday tasks on your own	13	2.2	2.3	1.15	1.24	87.9	84.5**	0.4	0.4
Engaging in your leisure activities	14	3.7	3.0	1.30	1.42	78.8	76.7	2.1	1.9
Putting on and taking off your clothes	15	2.2	1.9	1.13	1.20	88.7	86.1*	0.3	0.2
									
**Lifting/carrying**				**1.23**	**1.36**	**64.6**	**64.7**	**0.3**	**0.1**
Lifting an empty soda bottle crate from the floor	16	2.6	2.0	1.15	1.23	90.5	87.6**	1.7	1.3
Carrying shopping bags in your hands	17	2.4	1.8	1.23	1.31	82.1	82.1	1.1	0.6
Carrying a little sack/backpack on your shoulders or back	18	2.8	2.3	1.20	1.33	85.8	81.7**	1.8	1.7
Pushing and pulling with your arms	19	3.0	1.9	1.31	1.43	76.0	75.8	1.1	1.1
Cleaning your house	20	3.0	2.1	1.33	1.50	75.2	72.8	1.6	1.6
Washing your clothes	21	3.3	2.9	1.16	1.29	88.6	83.9***	1.3	1.6
									
**Sitting**				**1.10**	**1.19**	**87.0**	**82.2**	**0.1**	**0.1**
Sitting on a kitchen chair	22	2.5	1.8	1.08	1.16	93.2	89.7***	0.2	0.2
Riding as a passenger in a car	23	3.5	2.6	1.06	1.12	95.2	91.6***	0.2	0.2
Riding as a passenger on public transport	24	4.5	3.2	1.15	1.25	90.8	86.9**	2.1	1.9
									
**Managing**				**1.25**	**1.43**	**53.2**	**46.3**	**0.1**	**0.0**
Staying alert and being able to concentrate	25	2.7	2.2	1.26	1.40	77.3	72.7**	0.2	0.4
Working in groups	26	9.0	6.2	1.18	1.33	86.4	80.6***	1.4	1.3
Guiding others in their activities	27	9.3	7.1	1.19	1.34	86.7	80.6***	2.0	1.8
Managing everyday responsibility	28	3.3	2.9	1.15	1.30	87.6	80.0***	0.2	0.5
Managing everyday stress and strains	29	3.3	2.5	1.33	1.53	72.5	66.1***	0.4	0.7
Managing to take criticism	30	4.3	2.9	1.34	1.54	72.0	63.6***	0.9	0.5
Managing to control your anger and aggression	31	2.2	1.9	1.29	1.49	74.4	65.2***	0.5	0.3
									
**Cooperation/communication**				**1.18**	**1.32**	**58.7**	**49.8**	**0.0**	**0.1**
Remembering things	32	2.5	1.9	1.42	1.67	63.5	55.3***	0.5	0.3
Understanding spoken messages	33	2.7	2.1	1.21	1.39	81.6	71.2***	0.3	0.1
Understanding written messages	34	2.5	1.9	1.07	1.16	94.0	88.4***	0.3	0.2
Speaking	35	2.3	1.9	1.07	1.17	93.7	87.6***	0.0	0.1
Participating in a conversation with many people	36	2.6	2.1	1.19	1.35	84.3	77.4***	0.7	0.5
Using the telephone	37	1.9	1.5	1.07	1.15	94.2	90.9***	0.2	0.4
									
**Senses**				**1.05**	**1.09**	**94.7**	**91.3**	**0.0**	**0.0**
Watching television	38	2.0	1.6	1.05	1.10	96.1	93.0***	0.0	0.1
Listening to the radio	39	2.0	1.9	1.04	1.09	96.8	94.0***	0.3	0.1
									
**Total score**				**1.20**	**1.31**	**33.1**	**30.6**	**0.0**	**0.0**

^a^End effects for the NFAS-4 and NFAS-5 are compared, * p < 0.05; ** p < 0.01; *** p < 0.001

Item responses were skewed towards no difficulty for both versions (Table 2). The percentage of respondents reporting no difficulty for all 39 items was 33.1% in the NFAS-4 and 30.6% in the NFAS-5. In the general the NFAS-4 items had larger floor and ceiling effects than NFAS-5 items; some differences were statistically significant (p < 0.05) (Table 2). The third response alternative in NFAS-4 and the fourth in NFAS-5 had exact the same wording, "much difficulty", but the percentage response was lower in NFAS-5 than in NFAS-4 for 24 items.

### Scaling assumptions

All items in both versions met the 0.40 criterion for item-total correlation with the exception of the two items in the "senses" domain in NFAS-4 (Table 3). In all domains, item-total correlation coefficients were higher within the NFAS-5 than within NFAS-4, and this difference was significant for 35 items.

**Table 3:** Mean item-total correlation and Cronbach's alpha for domain scores in the NFAS-4 and the NFAS-5 (N = 3325)

	**Mean item-total correlation**		**Cronbach's alpha**^a^	
	**NFAS-4**	**NFAS-5**	**NFAS-4**	**NFAS-5**
**Walking/standing**	0.74	0.79	0.91	0.93***
**Holding/picking**	0.55	0.65	0.82	0.88***
**Lifting/carrying**	0.70	0.77	0.89	0.92***
**Sitting**	0.53	0.60	0.66	0.74***
**Managing**	0.66	0.72	0.87	0.91***
**Cooperation/communication**	0.60	0.66	0.81	0.85***
**Senses**	0.27	0.53	0.69	0.69
				
**Total scores**	0.62	0.70	0.95	0.96**

^a ^Cronbach's alpha values for NFAS-4 and NFAS-5 are compared, * p < 0.05; ** p < 0.01; *** p < 0.001

All items, except four in the NFAS-4 and one in the NFAS-5, met the item-discriminant validity criterion. Cronbach's alpha for two of the NFAS-4 and one of the NFAS-5 domains just failed to meet the 0.70 criterion (Table 3). Cronbach's alphas were significantly higher for NFAS-5 across the first six domains and the total score.

### Construct validity

For both versions, scores from conceptually related domains of NFAS correlated higher than scores of unrelated domains (Table 4). The NFAS-5 produced the largest correlations between domains and between domains and total scores, which was significant (p < 0.05) for 15 items and four domains.

**Table 4:** Correlation^a ^between NFAS, COOP/WONCA, GHQ-20 and Work ability for the NFAS-4 and the NFAS-5 (N = 3325)

**NFAS-4**	**Norwegian Function Assessment Scale**							**COOP/WONCA**			**GHQ-20**	**Work ability**
N = 1620	**Walk./stand.**	**Hold./pick.**	**Lift./carry.**	**Sitting**	**Manag.**	**Coop./Comm.**	**Senses**	**Phys. fitness**	**Feelings**	**Overall health**		

Walking/standing								**0.46**	0.30	0.58	0.36	0.50
Holding/picking up things	**0.67**							**0.38**	0.32	0.53	0.37	0.52
Lifting/carrying	**0.65**	**0.69**						**0.40**	0.33	0.54	0.39	0.50
Sitting	**0.51**	**0.53**	**0.51**					0.26	0.26	0.40	0.29	0.37
Managing	0.46	0.49	0.49	0.38				0.26	**0.61**	0.58	**0.62**	0.42
Cooperation/communication	0.37	0.40	0.39	0.26	**0.66**			0.26	**0.42**	0.45	**0.46**	0.34
Senses	0.25	0.26	0.27	0.22	0.24	0.33		0.11	0.16	0.20	0.18	0.20
												
Total scores	0.77	0.75	0.76	0.52	0.79	0.69	0.29	0.46	0.50	**0.69**	0.56	**0.56**

**NFAS-5**	**Norwegian Function Assessment Scale**							**COOP/WONCA**			**GHQ-20**	**Work ability**
N = 1705	**Walk./stand.**	**Hold./pick.**	**Lift./carry.**	**Sitting**	**Manag.**	**Coop./comm.**	**Senses**	**Phys. fitness**	**Feelings**	**Overall health**		

Walking/standing								**0.51**	0.25	0.57	0.36	0.51
Holding/picking up things	**0.73**							**0.41**	0.27	0.54	0.37	0.56
Lifting/carrying	**0.73**	**0.74**						**0.44**	0.28	0.55	0.40	0.58
Sitting	**0.59**	**0.60**	**0.63**					0.34	0.24	0.43	0.32	0.41
Managing	0.51	0.54	0.54	0.48				0.29	**0.56**	0.59	**0.61**	0.46
Cooperation/communication	0.43	0.47	0.44	0.40	**0.72**			0.28	**0.42**	0.48	**0.47**	0.38
Senses	0.30	0.34	0.32	0.33	0.36	0.42		0.19	0.18	0.27	0.25	0.26
												
Total scores	0.76	0.76	0.76	0.60	0.83	0.76	0.38	0.45	0.46	**0.67**	0.55	**0.57**

^a^Spearman's correlation

For all correlation coefficients: p < 0.001.

Bold numbers indicate apriori hypothesized associations with high correlation coefficients.

NFAS scores correlated higher with conceptually corresponding aspects of the COOP/WONCA, GHQ and Work Ability than with non-corresponding aspects for both versions (Table 4). The Sitting and Senses domains had relatively low correlations with these items or scales. The correlation coefficients were similar for the two versions. With only one exception, all the correlations hypothesized as being high, were over 0.40, indicating that the same construct was being measured by the NFAS and the external standard.

Both versions discriminated between persons anticipated to report different levels of functional ability, including persons with disability pension or medical rehabilitation, persons reporting sickness absence, and persons with physical versus mental symptoms (Table 5).

**Table 5:** Domain scores for different groups of the study population for the NFAS-4 and the NFAS-5 (N = 3325)

	**NFAS-4**						**NFAS-5**					
	**Disability pension/rehab.**	**All others**	**Sickness absence**	**No sickness absence**	**Phys. probl. only**	**Mental probl. only**	**Disability pension/rehab.**	**All others**	**Sickness absence**	**No sickness absence**	**Phys. probl. only**	**Mental probl. only**
N	196	1414	425	644	603	57	190	1500	461	701	641	76
**Walking/standing**	1.66	1.19***	1.22	1.09***	1.20	1.10*	2.13	1.28***	1.34	1.12***	1.33	1.11***
**Holding/picking**	1.39	1.11***	1.15	1.04***	1.10	1.05	1.74	1.16***	1.18	1.06***	1.18	1.10**
**Lifting/carrying**	1.64	1.18***	1.24	1.09***	1.20	1.06**	2.15	1.26***	1.33	1.11***	1.29	1.12**
**Sitting**	1.34	1.07***	1.09	1.03***	1.08	1.03	1.64	1.13***	1.16	1.05***	1.14	1.05
**Manag.**	1.59	1.20***	1.30	1.13***	1.16	1.39***	2.04	1.35***	1.45	1.23***	1.31	1.55*
**Coop./comm.**	1.36	1.15***	1.18	1.09***	1.12	1.29***	1.69	1.27***	1.31	1.19***	1.26	1.33
**Senses**	1.16	1.03***	1.04	1.01***	1.03	1.03	1.24	1.08***	1.09	1.04*	1.07	1.07
**Total scores**	1.49	1.15***	1.20	1.08***	1.15	1.16	1.91	1.24***	1.30	1.13***	1.25	1.22

* p < 0.05; ** p < 0.01; *** p < 0.001; Mann Whitney U-test

For both versions, a decline in physical functional ability was significantly associated with increasing age (p < 0.05). With one exception, males reported significantly better functional ability (p < 0.001) for both versions. With the exception of the Senses domain for the NFAS-4, a significant education gradient was found for both versions (p < 0.001).

Applying age-stratified analyses, the results for data quality, scaling assumptions and construct validity remained stable.

## Discussion

Both versions demonstrated low levels of missing data and skewed response distribution, but the NFAS-4 had more missing values and larger end effects than NFAS-5. The NFAS-5 demonstrated better internal consistency and item-discriminant validity than the NFAS-4, although the results were acceptable for both versions. All a priori hypotheses were met, which strongly supports the construct validity of the scale for both versions. Both versions discriminated similarly well between groups with different levels of health status and between known groups in the population.

### Data quality

The response rates and the low levels of missing data show that both versions of the NFAS are acceptable to the population. A few items had a high percentage of missing values, which is probably because there was no "not applicable" option. Significantly less missing data for the NFAS-5 than the NFAS-4 is some indication that the respondents found it easier choosing a suitable response from the five-point scale. This finding is supported by Nagata et al. [27], who compared feasibility of health measurement response scales using four, five and seven categories and a visual analog scale. The level of missing data was least and the responder preference was highest, for the five-point scale version.

Since the NFAS data are skewed towards higher levels of functioning, the larger end effects for NFAS-4 have to be considered when the instrument is used to discriminate between different levels of functioning or to assess changes in functioning over time. It is likely that NFAS-4 will not be as responsive to changes in functioning, simply because it has fewer response options that individuals can use to indicate that their functioning has changed.

It might be anticipated that the response alternative, "much difficulty", along with the two end categories would show similar percentages in the two versions. This was not found. Hence, the responses did not seem to be affected by the wording or anchoring of the response alternatives.

### Internal consistency and validity

The internal consistency values were similar to widely used instruments including the SF-36 [28-33] and the NHP [15]. Our item-other domain correlation coefficients were comparable with other study results using the SF-36 in a study including rheumatoid arthritis patients [34] and a population study [29].

Regarding construct validity, different time perspectives in the questioning for the different scales could influence possible associations since Work Ability concerns today, NFAS last week, COOP/WONCA and GHQ the last two weeks. However, all a priori hypotheses correlation coefficients met the 0.4 – 0.8 standard. Other studies have obtained similar correlation coefficients between NHP and SF-36 scales [15,34] or between SF-36 scale scores and comparable item or domain scores from other questionnaires [32,35]. Regarding the ability to discriminate between groups with different levels of health status, comparable results were found for the SF-36 [30-33,35]. A gender difference was found in several studies [28,30-32,35-37], but not all [33,38]. The finding of a physical age gradient is supported by several studies [28,32,33,35-38], and an education gradient has also been found in previous research [28,30,31,35,38].

The NFAS-5 demonstrated somewhat higher internal consistency and item-discriminant validity values compared to the NFAS-4. The majority of this difference could probably be attributed to the fact that correlation between true values and scale scores increase with the number of response options [12], but it is not known whether this explains the whole difference in correlation coefficient values.

### Future applications of the NFAS

The items in the NFAS are derived directly from the activities/participation dimension in the ICF. The ICF use a five-point scale for their qualifiers and the clinical checklists. This supports the use of the NFAS-5. The NFAS-5 had lower levels of missing data than the NFAS-4 which may indicate higher responder acceptability. The NFAS-5 generally performed better than the NFAS-4 in relation to the psychometric tests. Therefore the five-point scale is recommended in future applications of the NFAS. The main drawback in changing to a new response format is that it precludes direct comparisons between previous and new research. However, following our study results, we believe that the evidence supports changing the NFAS response format to a five-point scale.

### Strengths and limitations

This study' strengths include the randomised design, the large study sample, the good data quality and the thorough testing of validity against other standards. The moderate response rate and that all data is self-reported, represent study limitations. An external, unrelated variable would have strengthened validity assessment. With the present study design it was not possible to ask the respondents about their preferences [10] or to determine the sensitivity to change, the responsiveness of the scale. However, the low mean missing values may indicate acceptability among respondents.

## Conclusion

The data quality of NFAS is high with acceptable internal consistency and good construct validity. In choosing between the four-point and the five-point scale, it should be noted that while construct validity and discriminative ability are comparable, both data quality, internal consistency and discriminative validity suggest that the five-point scale is to be preferred in future applications of the NFAS.

## Abbreviations

GHQ-20: The General Health Questionnaire-20 items; ICF: The International Classification of Functioning, Disability and Health; NFAS: The Norwegian Function Assessment Scale; SF-36: The generic Short Form 36-item Health Survey

## Competing interests

The author(s) declare that they have no competing interests.

## Authors' contributions

NØ planned and designed the study, performed some of the statistical analysis, drafted the manuscript and coordinated the study. PG participated in the planning and design of the study, interpretation of the results and in drafting the manuscript. AG helped in the interpretation of the results and participated in drafting the manuscript. JSB performed most statistical analysis and reviewed the manuscript. FAD assisted statistical analysis and reviewed the manuscript. BN participated in planning and designing the study, collected the data and participated in drafting the manuscript. SB planned and designed the study, participated in the interpretation of results and in drafting and revising the manuscript. All authors read and approved the final manuscript.
